# IgG N-glycan Signatures as Potential Diagnostic and Prognostic Biomarkers

**DOI:** 10.3390/diagnostics13061016

**Published:** 2023-03-07

**Authors:** Benjamin S. Haslund-Gourley, Brian Wigdahl, Mary Ann Comunale

**Affiliations:** 1 Department of Microbiology and Immunology, Drexel University College of Medicine, Philadelphia, PA 19129, USA; 2 Institute for Molecular Medicine and Infectious Disease, Drexel University College of Medicine, Philadelphia, PA 19129, USA

**Keywords:** immunoglobulin G, IgG, N-glycan, diagnostic, prognostic, glycosylation, glycopeptide

## Abstract

IgG N-glycans are an emerging source of disease-specific biomarkers. Over the last decade, the continued development of glycomic databases and the evolution of glyco-analytic methods have resulted in increased throughput, resolution, and sensitivity. IgG N-glycans promote adaptive immune responses through antibody-dependent cellular cytotoxicity (ADCC) and complement activation to combat infection or cancer and promote autoimmunity. In addition to the functional assays, researchers are examining the ability of protein-specific glycosylation to serve as biomarkers of disease. This literature review demonstrates that IgG N-glycans can discriminate between healthy controls, autoimmune disease, infectious disease, and cancer with high sensitivity. The literature also indicates that the IgG glycosylation patterns vary across disease state, thereby supporting their role as specific biomarkers. In addition, IgG N-glycans can be collected longitudinally from patients to track treatment responses or predict disease reoccurrence. This review focuses on IgG N-glycan profiles applied as diagnostics, cohort discriminators, and prognostics. Recent successes, remaining challenges, and upcoming approaches are critically discussed.

## 1. Introduction

Discriminating between states of health and disease relies on clinicians developing a differential list of candidate diagnoses from a patient’s symptoms and presentation. The differential diagnosis can be confirmed by testing the patient’s serum, stool, blood, saliva, urine, spinal fluid, or tissue biopsy using clinical laboratory tests, providing a test is available [1]. Many diseases have highly sensitive and specific diagnostics based on directly detecting the infectious agent, immune marker, or cellular response to the disease [2]. Other diseases lack sensitive diagnostics due to low-bacterial titers, nonspecific symptom presentation, and lack of specific diagnostic biomarkers [3,4,5,6]. In addition to diagnosis, the prognosis of a patient’s response to treatment can also be challenging [7,8].

“Omics” is a method used to classify research approaches that can measure biological molecules in a high-throughput manner. The emergence of the omics field began with the completion of the mapping and sequencing of the human genome. Commonly used methods of biomarker discovery include genomics, proteomics, and metabolomics; however, glycoproteomics and glycomics are emerging as important clinical diagnostic tools. Many circulating proteins are glycosylated and have been demonstrated to change during disease states such as cancer [9,10,11]. One source of glyco-biomarkers, that is well studied and reflects the host’s immune response to disease over time, is the glycosylation of immunoglobulin G (IgG) [12]. This manuscript reviews the diagnostic and prognostic potential of the IgG glycan signature.

**Figure 1:**
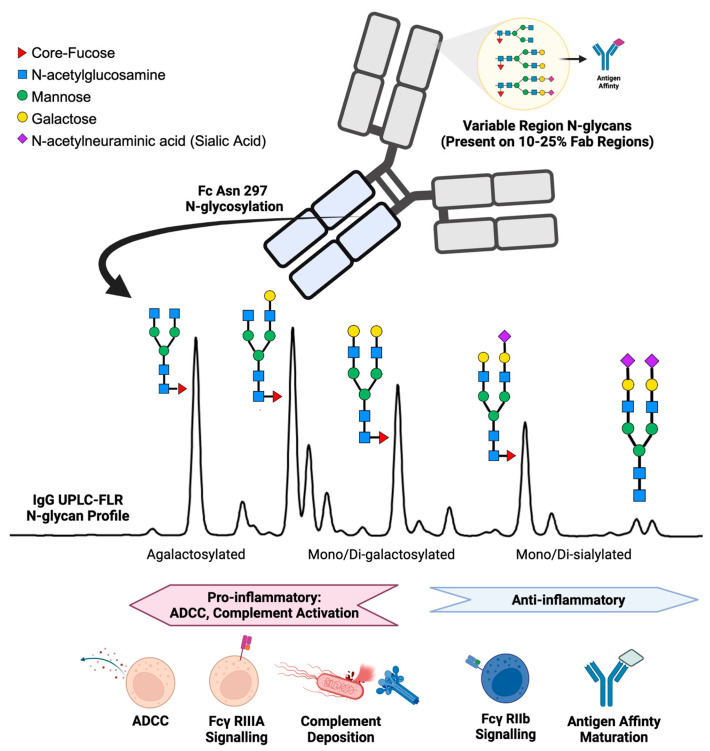
Summary of IgG N-glycan present on the variable region (Fab) and the constant heavy chain tail region (Fc) with associated immunologic functions.

IgG structure and function are affected by the presence of N-linked glycans. IgG N-glycan profiles consist of 24 different biantennary complex N-glycans that range in size and charge depending on the presence of N-acetylglucosamine, galactose, core-fucose, and sialic acid (Figure 1) [13,14]. IgG contains one conserved site of glycosylation on the Aspragine-297 on each of its two Fc heavy chains [15]. In addition, 10–25% of the variable region (Fab) of IgG also contains N-glycans, but accounts for a minor portion of the total N-glycosylation and plays a role primarily in antigen-affinity and serum half-life of the antibody [16,17,18]. During health, the post-translational addition of N-glycans to the Fc region of IgG yields a reproducible profile [19,20]. Immunologically, the IgG N-glycosylation of the Fc region plays a vital role in directing antibody-dependent cellular cytotoxicity (ADCC) and complement-mediated cytotoxicity [21,22,23,24]. ADCC is promoted by reduced fucose, galactose, and sialic acid content enabling the Fc portion of IgG to bind receptors such as Fcγ-RIIIa on immune cells to signal for activation [25,26,27,28]. In contrast, the addition of fucose, galactose, and sialic acids alters the conformation of the Fc region on IgG to promote inhibitory Fcγ-RIIb receptor binding, resulting in an anti-inflammatory response [29,30,31]. IgG Fc N-glycans promote downstream complement-mediated cytotoxicity by binding C1q, while increased IgG Fc sialic acid content inhibits complement deposition [32,33,34]. Further evidence of IgG N-glycan’s immunological importance is reflected by therapeutic monoclonal antibodies requiring glycoengineered afucosylated, agalactosylated Fc glycoforms to promote effective antibody function [35,36]. In addition, murine studies have demonstrated that IgG glycosylation driving autoimmune pathology can be modified enzymatically with EndoS, rendering disease-causing collagen-specific antibodies non-pathogenic in mice [37,38]. Moreover, when autoantibodies causing the disease neuromyelitis optica were treated with EndoS, the same antibodies became protective, serving as a potent therapeutic [39].

N-glycans decorating IgG are sequentially added by B lymphocyte glycosyltransferases (GTs) in a highly regulated fashion [40,41]. Expression of GTs in B lymphocytes is altered by a growing list of cytokines including INF-γ, TNF-α, IL-17A, T_H_17, IL-21, and IL-23 as well as environmental factors such as trans-retinoic acid and CpG oligodeoxynucleotide [42,43,44]. For example, Pfeifle et al. reported the IL-23-T_H_17 axis alters antigen-specific IgG N-glycans to contain more pro-inflammatory Fc content during rheumatoid arthritis. In a murine model, they demonstrated IL-23 activated T_H_17 cells to accumulate in germinal centers of lymph nodes and suppress the expression of sialyltransferase GTs in differentiating plasmablasts. As a result, the autoantibodies produced by plasmablasts contained lower sialic acid content. These pro-inflammatory autoantibodies contained higher levels of galactose and GlcNAc on the Fc region and induced downstream immune activation (ADCC and complement deposition), leading to symptomatic rheumatoid arthritis flairs [42]. Thus, extracellular stimuli can alter B cell GT expression, resulting in different IgG N-glycosylation profiles. The exact mechanisms controlling IgG N-glycosylation in humans are not fully elucidated at this time. However, during immune responses the plasmablasts, B cells, T cells, and other immune cells likely participate to induce a specific glycosylation profile on the responding IgG.

Changes in protein glycosylation can be rapid, making them feasible biomarkers of acute disease and prognosis. For example, in patients that underwent cardiac surgery, a consistent global serum glycome change was observed within the first 24 h. Importantly, in patients that had a systemic inflammatory reaction, specific IgG glycan signatures were associated with a higher rate of mortality [45]. Research is ongoing to determine the frequency and mechanism of N-glycan modification of circulating IgG. However, current data suggest that once in circulation, most IgG N-glycans are not enzymatically transformed, with the possible exception of sialylation [46,47]. Taken together, IgG N-glycan profiles reflect the state of a host’s immune response and hold great potential as sensitive biomarkers of health and disease. We highlight recent applications of IgG N-glycan profiling during inflammation, autoimmunity, and cancer and present the diagnostic and prognostic potential of IgG N-glycans.

## 2. IgG N-Glycans in Healthy Humans

Serum glycoproteins contain many different glycan moieties, but the overall protein glycosylation pattern for each protein remains constant in the healthy state. Total serum IgG N-glycosylation profiles from healthy humans fall within a specific range for each of the major N-glycan classes: 35% agalactosylated, 35% mono-galactosylated, 15% di-galactosylated, 10% mono- and di-sialylated, 10% bisecting, and 90% core-fucosylated [23,48]. Conversely, in disease glycosylation can be dynamic and reflect specific disease states.

The healthy IgG N-glycan profile often reflects an immune system without infection, cancer, or an autoimmune condition. Age impacts the IgG N-glycan profile; pediatric age ranges gain galactose into young adulthood [49] while older adults decrease galactose content [50,51,52]. Differences in IgG N-glycosylation also arise when comparing cohorts stratified by: males vs. females [53,54], diet [55], menopause [56,57], pregnancy [17], physical exercise [58], country of residence [59], and body mass index [60,61,62,63,64]. As the following sections will describe, the IgG N-glycan profile can shift dramatically beyond the confounding effects listed above when responding to infection, autoimmunity, or cancer. However, it is important to account for the innate differences in IgG N-glycan profiles when developing diagnostic algorithms.

## 3. IgG N-Glycans Altered during Infectious Disease

Infectious and inflammatory diseases are associated with antigen-specific IgG, IgG subclasses (IgG1-4), and total IgG N-glycosylation profile alterations. Characterizing IgG N-glycans during disease states compared to healthy control cohorts can accurately reflect the host’s immune status [65]. Moreover, longitudinally profiling a patient’s IgG N-glycosylation following diagnosis and during treatment can track patient immune responses [66]. This literature review has collected publications from 2012 to 2022 describing IgG N-glycans potential as a diagnostic or prognostic (Table 1). Initially, we expected to see similarities across many diseases, and as shown in the table, there are general trends of glycan classes increasing or decreasing. However, the combination of the changes in IgG glycosylation varies considerably across diseases.

### 3.1. IgG N-Glycans Altered during Viral Infections

Viruses require host machinery to replicate and can be classified depending on their replication strategy, tissue tropism, envelope, chronicity, and carcinogenic potential [106]. Viruses are incapable of glycosylation; however, many viral proteins contain N-linked glycans that are added by hijacking the host cell machinery. While the diagnosis of viral infections is mainly accomplished through the detection of viral loads [107], the prognosis of a patient and potential mechanisms promoting disease responses via IgG N-glycosylation has received attention in the literature in recent years.

### 3.2. Dengue Fever

Acute viral infections are known to alter IgG N-glycan profile in infected humans. Dengue (DENV) is a flavivirus that induces the host to produce antibodies that can drive pathology through antibody-dependent enhancement upon reinfection [75,108]. The glycosylation of IgG antibodies during a severe DENV re-infection offers a clear example of Fc N-glycans driving a disease phenotype. During severe DENV infections, IgG N-glycans profiles become proinflammatory—associated with lower fucosylation, more agalactose exposure, and lower sialylation [75]. As a result of the glycosylation, DENV antibodies produced during severe disease promote increased complement deposition and ADCC in humans. Severe DENV survival is strongly associated with a higher proportion of core-fucose on IgG1 in humans. In vivo functional experiments demonstrate that afucosylated IgG1 produced in response to DENV infection activates platelets and leads to significant thrombocytopenia due to FcγR IIIA pathway activation. In addition, maternal Dengue antigen-specific IgG fucosylation levels predict an infant’s susceptibility to a severe Dengue infection [76,77]. Thus, IgG N-glycan profiles can predict susceptibility to severe DENV infection and play a role in disease pathogenesis.

### 3.3. Influenza and RSV

Influenza viruses are associated with seasonal epidemics in humans, leading to more than 100,000 deaths annually [109]. Annual influenza vaccinations are deployed to protect older and immunocompromised individuals within the population. While a plethora of research focuses on the effect of influenza vaccination on serum IgG glycosylation and therapeutic IgG antibody glycosylation, there are few studies that examine the host IgG glycan response to a natural infection. One study looked at patients hospitalized for influenza infection and determined that the patient serum IgG N-glycosylation exhibited increased sialic acid while also decreasing core-fucose content [68]. The decreased fucosylation was associated with heightened ADCC while the increase in sialic acid was associated with antigen affinity maturation.

Respiratory Syncytial Virus (RSV) is a source of severe lower respiratory tract infections in young children, leading to over 100,000 estimated worldwide deaths annually [110]. Antigen-specific IgG from infants was examined for Fc glycosylation, revealing that the degree of fucosylation correlates with the natural killer (NK) cell activation [69]. Furthermore, RSV-specific IgG isolated from severe RSV infants induces lower levels of IFN-γ compared to healthy age-matched controls. This work suggests that RSV-specific IgG glycosylation is a potential correlate of protection to assess RSV vaccine response and may denote infants at greater risk for developing severe RSV infection.

### 3.4. SARS-CoV-2

SARS-CoV-2 (COVID-19) has impacted the world significantly since its outbreak in late 2019, killing more than 14 million between 2020 and 2021 [111]. RT-PCR and rapid antigen tests have helped to accurately diagnose patients early in the disease progression [112], yet predicting a patient’s outcome and hospitalization requirements remain challenging. A case–control study determined that during severe disease, IgG N-glycans lost significant levels of fucose and had lower levels of sialic acid content compared to patients with a mild case of COVID [28]. Because IgG N-glycans can promote complement deposition and ADCC, the N-glycan profile could identify patients in a highly proinflammatory state and associated with a heightened risk for a cytokine storm. Other studies have confirmed a lower level of sialylation and galactosylation with a concomitant increase in agalactose content associated with hospitalized COVID-19 patients who did not survive [68,70].

In addition to total IgG N-glycan profiles, antigen-specific IgG N-glycan profiles has been isolated and analyzed, affording a more detailed perspective of the B-cell response to COVID. When anti-spike protein IgG1 was analyzed, there was a significant loss of galactose and sialyation with concurrent increased bisecting GlcNAc glycans in patients [71]. The spike-specific IgG1 N-glycosylation patterns could predict, at the time of hospital admission, which patients would require ICU admission in the following days. COVID-19 severity has been linked to an overactivation of the ADCC pathway by afucosylated anti-spike IgG N-glycans [72]. Moreover, a longitudinal study of severe- vs. mild-COVID, that included four European cohorts, identified a loss of sialic acid and bisecting N-glycans, and a rise in agalactose content in the total IgG profile of severe COVID patients [73]. These results were affirmed by another study that also found lowered sialylation and more agalactose content on IgG was associated with a poor prognosis leading to an AUC of 0.72 [74]. Taken together, these results support the notion that IgG N-glycans play a role in the pathogenesis of serve COVID and are a valuable tool to triage patients who will require more intense medical support during this viral respiratory illness.

### 3.5. HIV

Chronic viral diseases such as HIV can impact the health of the patient over time and are monitored for disease progression. Understanding human immunodeficiency virus (HIV) progression and viral rebound is important in developing treatment and vaccination strategies [113]. HIV rebound during antiretroviral therapy (ART) is strongly associated with patients’ levels of di-galactosylated and agalactosylated IgG in two geographically distinct cohorts [97,98]. IgG N-glycan profiles also differed between adolescents with HIV that progressed to more severe immunocompromised states compared to HIV non-progressors [99]. Pediatric HIV non-progressors exhibited IgG Fc N-glycosylation associated with a more potent effector function. Both progressor and non-progressor HIV pediatric cohorts exhibited more sialyation on gp120-specific IgG Fc regions. This sialyation was associated with increased broad neutralizing antibody (bnAb) breadth and suggests that increased sialyation on IgG may promote affinity maturation in children as compared to the lower bnAb production in adults. The authors go on to postulate IL-21 stimulation of B cells by T follicular helper cells could promote increased sialyation and galactosylation of IgG [44]. Thus, IgG N-glycans can add clinical value when predicting viral rebound and driving antigen-specific responses to HIV infection.

### 3.6. HBV

Hepatitis caused by the hepatitis B virus (HBV) is highly prevalent and leads to an estimated 820,000 deaths from liver fibrosis and progression to liver cancer [114,115]. Patients require monitoring over their lifetime for disease progression. However, effective monitoring requires costly ultrasounds along with bloodwork every six months. Diagnostics such as the GlycoTest have identified increased fucosylation on other serum glycoproteins leading to liver cancer and are currently in clinical trials [116]. Previous studies of IgG N-glycosylation during Chronic hepatitis B are associated with increased agalactose content on the Fc region of IgG [100]. Moreover, the aberrant glycosylation of IgG was reversed in noncirrhotic chronic hepatitis B patients following antiviral therapy. Recent studies of IgG N-glycans have identified specific changes in IgG subclasses 1–4 that discriminate cohorts of healthy controls, early fibrosis, and significant fibrosis with sensitivities of 92%, 84%, and 94%, respectively [104]. Taken together, IgG N-glycans are a valuable resource to determine the patient’s prognosis, stage of disease, and treatment response while avoiding the need for invasive liver biopsies.

## 4. IgG N-Glycans Altered during Bacterial and Parasitic Infections

Few bacterial or parasitic infections have been analyzed for their host response of IgG N-glycosylation. This may be due to many bacterial infections being acute inflammatory conditions that are often identified and treated with the appropriate antibiotic rather than chronic infections [117]. Although bacterial latency and chronicity in humans is less common in bacterial infections than with viruses, they still represent an enormous global disease burden and adversely impact patient quality of life.

### 4.1. Tuberculosis

Tuberculosis (TB) is first bacterial infection to have a comprehensive serum IgG N-glycosylation analysis completed. TB is a bacterial infection that can lead to chronic, life-long infection with one out of three people currently infected with TB globally [118]. Biomarkers of active versus latent TB have been elusive, resulting in patients with active, contagious disease not being effectively triaged for treatment. IgG N-glycans were used to discriminate between a latent TB infection and an active TB infection by tracking the loss of galactose and sialic acid content on the IgG Fc region of patients with active TB in two separate cohorts [94]. IgG N-glycans reverted to the less inflammatory, more galactosylated profile in patients who had received treatment for TB [95], indicating usefulness in determining disease resolution. Lastly, measuring the ratio of agalactose to galactosylated IgG N-glycans detected TB patients compared to healthy controls with a sensitivity of 76% and a specificity of 71% [96].

### 4.2. Lyme Disease

Patients with acute Lyme disease (LD) are often seronegative early during the disease and risk disease progression if not treated with antibiotics promptly. Total IgG N-glycosylation was significantly altered in a cohort of acute Lyme disease patients [67]. Surprisingly, the N-glycans displayed reduced agalactose and increased di-galactose content during the acute stage which led to a sensitivity of 75%, and specificity of 100% for acute LD discrimination from healthy controls. Moreover, in patients who received treatment and returned to donate serum after 70–90 days of convalescence, the N-glycans on IgG could be used to discriminate between treated and acute Lyme disease with a sensitivity and specificity of 100%. This suggests that IgG N-glycans can differentiate disease states during Lyme disease and may identify patients successfully responding to antibiotic treatment.

### 4.3. Meningococcus

Pediatric patients with meningococcal sepsis are at a heightened risk of morbidity and mortality. Prognostic indicators of disease outcomes are of great interest and could aid to initiate appropriate treatments [119]. Recent work identified that IgG1 fucosylation was reduced and bisection was increased in patients aged 0 to 3.9 years of age compared to healthy age-matched controls [52]. Moreover, when meningococcal septic patients aged 0–3.9 years of age were stratified into severe and non-severe cohorts, hybrid type N-glycans on IgG1 and IgG2/3 were significantly reduced while all IgG isotypes also contained significantly lower sialyation. The IgG Fc glycosylation is also associated with other inflammatory markers including thrombocyte levels, fibrinogen, and the cytokine IL-6. Hybrid structures on IgG2/3 had significantly positively correlated relationships between CRP and leukocyte levels. This work demonstrates the severity of meningococcal sepsis can be predicted using the glycosylation of the Fc portion of IgG. More work is needed to confirm these data in a larger cohort and potentially combine IgG glycosylation with other markers of severity such as IL-6 or C-reactive protein.

### 4.4. Malaria

Malaria is a protozoan parasitic infection spread to humans from the bite of an infected mosquito, costing hundreds of thousands of mostly young children’s lives annually in endemic areas [120]. When Larsen et al. characterized the glycosylation of total IgG from people in malaria-endemic regions, they detected high levels of core-fucose, similar to non-malaria endemic region total IgG fucosylation. When they isolated malaria antigen-specific IgG1 developed from natural infection, they detected significantly lower levels of core-fucose [105]. In contrast, antigen-specific IgG1 produced in response to a malarial subunit vaccination did not induce lower core-fucose content on antigen-specific IgG1. The group demonstrated that only afucosylated IgG1 could induce ADCC against the malarial antigen, indicating the need to design malarial vaccines that better mimic natural infection to produce effective IgG with lower core-fucose content.

## 5. IgG N-Glycans Altered during Autoimmune and Inflammatory Diseases

Autoimmune disease is characterized by the loss of humoral tolerance and the appearance of circulating autoantibodies [121]. The development of autoimmunity is associated with a dysregulated adaptive immune system; thus the dynamic role of IgG glycosylation lends itself to tracking disease progression and treatment response in autoimmune diseases. IgG has been implicated in many autoimmune diseases as a major driver of disease phenotype. Understanding how the glycosylation of these pathologic immunoglobulins will be crucial to predicting disease relapse and curating innovative therapies [121].

### 5.1. Guillain–Barré Syndrome

Guillain–Barré syndrome (GBS) often occurs following acute viral infection and appears to be driven by auto-reactive antibodies directing the immune system to attack the peripheral nervous system [122]. Fokkink et al. determined patients with GBS had lowered galactose and sialic acid content on IgG1 and IgG2 when compared to healthy controls [78]. Patients with lower galactose and sialic acid of the IgG1 and 2 correlated to a higher frequency of mechanical ventilation and a longer time to recover. A common treatment for GBS is intravenous IgG (IVIG) from pooled healthy donors. This IVIG therapy for GBS was demonstrated to return IgG1 and IgG2 galactose and sialic acid content to normal levels and reduce inflammation [123]. IVIG treatment for GBS provides an example of IgG N-glycans driving disease and a potential therapy to help the host re-establish normal glycan profiles on IgG.

### 5.2. Systemic Erythematosus Lupus

One of the most well-known autoimmune conditions is systemic erythematosus lupus (SLE). SLE is a chronic autoimmune disease with a relapsing and remitting course characterized by excessive production of autoantibodies [124]. Total IgG N-glycan profiles isolated from a cohort of SLE patients revealed specific changes in fucosylated bisecting N-glycans that were combined with age and sex demographics to differentiate between healthy controls and SLE patients with an AUC of 0.84 [79]. This study also performed genome-wide analysis and identified altered expression of glycosyltransferases that aligned with the observed alteration in the IgG N-glycan profile.

### 5.3. Crohn's Disease and Ulcerative Colitis

Autoimmune diseases can present with similar or overlapping symptoms, leading to difficulty in accurately diagnosing and initiating the correct therapy. Ulcerative colitis (UC) and Crohn's disease (CD) are two common inflammatory bowel diseases driven by aberrant host immune response to luminal gut microbiota [125]. Discriminating between UC and CD is sometimes hard to distinguish without invasive testing. IgG N-glycan profiles were analyzed from a large cohort of UC and CD patients. The results of this study identified both UC and CD patients had increased levels of IgG agalactosylation. However, core-fucosylation was higher for CD versus; healthy controls while core-fucosylation of UC was much lower compared to healthy controls. Thus, the severity of the disease was estimated by the agalactose content while core-fucose content could discriminate between UC and CD patients with an AUC 0.75–0.77 [80].

### 5.4. Immune Thrombocytopenia

Immune thrombocytopenia (ITP) is an autoimmune condition associated with a low platelet count, risking severe bleeding [126]. ITP frequently arises following a febrile illness, suggesting an “over-activation” of the humoral immune response to an initial inflammatory condition. Conflicting reports confound the ability of IgG glycosylation to track disease treatment in ITP. One study reported that IgG N-glycan profiles did not differ between healthy control compared to ITP patients during an acute disease episode [127]. However, another group reported that IgG N-glycans from acute ITP patients exhibited lower core-fucosylation, which is strongly associated with ADCC promotion [81]. The Wang et al. group reported a decrease in the F(6)A2BG2 N-glycan discriminated between ITP patients and healthy control patients with an AUC of 0.96.

### 5.5. Idiopathic Membranous Nephropathy

Early stage urologic disease lacks specific biomarkers and often requires invasive testing or biopsies for the diagnosis of disease [128]. For example, idiopathic membranous nephropathy (IMN) reflects immune-complex driven destruction of the kidney glomerulus that can progress to end-stage renal disease [129]. IgG4 N-glycosylation profiles from IMN patients contained significantly lower galactose and increased core-fucose N-glycans compared to healthy controls and related kidney pathologies [83,84]. This recent work demonstrated a potential lectin complement-dependent mechanism of IMN pathology while also highlighting IgG N-glycans’ potential as biomarkers to stratify disease states from closely related kidney pathologies.

In addition, another group applied supervised machine learning to IgG N-glycan profiles collected from patient cohorts of hormone-sensitive prostate cancer, castration-resistant prostate cancer, renal cell carcinoma, upper urinary tract urothelial cancer, bladder cancer, germ cell tumors, benign prostatic hyperplasia, urosepsis, and urinary tract infection as well as healthy volunteers. Although these renal conditions share inflammatory phenotypes, an AUC of 0.78–1.0 was obtained when an IgG N-glycan profile scoring system was employed to provide a probability of the presence of each specific urological disease [86]. This work demonstrates the specificity of various N-glycan alterations on IgG resulting from different infectious and inflammatory conditions, and one day could reduce the number of invasive procedures required to confirm a diagnosis.

### 5.6. Vasculitis

Another autoimmune disease driven by antibodies is vasculitis. Vasculitis of the small to medium-sized blood vessels driven by autoantibodies binding proteinase-3 (PR3) or myeloperoxidase (MPO) proteins associated with neutrophil activation [130]. Predicting disease relapse is important to initiate therapy to prevent further damage to host vasculature. Samples from the vasculitis granulomatosis with polyangiitis (GPA) were analyzed for changes in total IgG N-glycosylation preceding disease relapse. IgG1 N-glycan profile comparisons identified a significant decrease in sialic acid and galactose in relapsing patients compared to non-relapsing patients, leading to an AUC of 0.65 [22].

### 5.7. Chronic Inflammatory Airway Diseases

Chronic inflammatory airway diseases such as severe asthma and sarcoidosis require invasive procedures for diagnosis [131]. Assessing disease severity and determining patient prognosis for chronic inflammatory lung diseases is crucial to initiate sufficient treatment. Severe asthma patient IgG4 and IgG1 Fc N-glycans were found to contain the least sialic acid and galactose compared to healthy controls, while patients with sarcoidosis were found to have a similar trend of glycosylation but to a lesser degree [82]. Furthermore, age-controlled IgG N-glycans could discriminate patients with sarcoidosis and severe asthma from healthy control patients with an AUC of 0.83. Thus, the ratio of agalactosylated to galactosylated IgG N-glycans from chronic inflammatory lung disease patients reflects the pro-inflammatory state of the patient and could be used to inform patient treatment and track treatment efficacy.

### 5.8. Amyotrophic Lateral Sclerosis

While not traditionally considered an autoimmune disease, amyotrophic lateral sclerosis (ALS) is a fatal neurodegenerative disease caused by the degeneration of motor neurons [132]. IgG was identified in the motor cortex of ALS patients and initial serum N-glycan analysis from ALS patients described low levels of fucosylated N-glycans. Subsequently, serum-derived IgG Fc N-glycans were discovered to have 2-fold higher concentrations of the afucosylated, bisecting, di-galactosylated N-glycan A2BG2 compared to healthy controls [101]. Moreover, IgG from ALS patients was associated with a higher level of ADCC-mediated cellular lysis in vitro. This result was suggested as a mechanism to promote neuronal damage during disease progression. This work highlights the A2BG2 IgG N-glycan as a potential biomarker for ALS while also providing insight into a mechanism of neuronal damage. More recently, IgG was isolated from cerebrospinal fluid, the associated N-glycan profile revealed a significant increase in mono- and di-galactosylated N-glycans were observed in the ALS patient cohort compared to healthy controls [102]. The summation of the galactose content was used to obtain an AUC of 0.79 discriminating between ALS patients and healthy controls. This was compared to the benchmark biomarker of ALS, phosphoneurofilament heavy chain which obtained an AUC of 0.78. However, this recent report did not observe the increased levels of A2BG2 as previously reported.

### 5.9. Parkinson’s Disease

Parkinson’s disease is a neurological pathology associated with the destruction of the dopaminergic neurons in the substantia nigra resulting in rigidity, slowness, and tremor [133]. It is essential to diagnose Parkinson’s disease and monitor progression using non-invasive tests. Therefore, the IgG N-glycome was analyzed as a candidate for biomarkers which yielded four N-glycans that when combined into a logistic regression model, provided a sensitivity of 87.2% and a specificity of 92.2% [103]. It is suggested that lack of monosiaylated N-glycans disinhibited ADCC activity and could promote the pathogenesis of Parkinson’s destruction of dopaminergic neurons. More research is needed to determine if this discriminatory N-glycan panel holds true for a larger cohort and if the mechanism of increased ADCC is a factor driving disease pathogenesis.

## 6. IgG N-Glycans Altered in Cancer

Early diagnosis of cancer is crucial for positive clinical outcomes, particularly in cancers not easily identified through annual screenings or those located in soft tissues that lack a clear diagnostic presentation [134]. IgG N-glycan profile analysis has been demonstrated as a noninvasive method to identify various malignancies in humans. Below are recent reports applying IgG N-glycans to discriminate between cancer stages and healthy controls.

### 6.1. Breast and Cervical Cancer

Many cancers in females are challenging to diagnose early in disease progression, and novel biomarkers could increase survival rates by initiating treatment earlier [135]. Stage II breast cancer patients were differentiated from healthy controls using the increased content of IgG N-glycans FA2G0 (AUC of 0.96) and FA2BG0 (AUC of 0.92) [87]. In addition, endometrial cancer (EC) patient IgG N-glycans in combination with BMI and age differentiated EC patients from healthy controls with an AUC of 0.87. This discrimination between healthy control and EC patients was driven by lower galactose and sialic acid content [88]. Serum IgG fucosylation decreased in women with cervical intraepithelial neoplasia I (CIN I). Females with cervical cancer were detected using a high-throughput enzyme-linked lectin assay (ELLA) [89]. Healthy controls were discriminated from CIN I patients using a fucose-quantitating ELLA with a sensitivity of 73% and a specificity of 62%. Meanwhile, the IgG fucose content discriminated healthy controls from cervical cancer patients with a sensitivity of 87% and a specificity of 72%.

### 6.2. Thyroid Cancer

Many cancers present in both males and females also lack sensitive early biomarkers and would benefit from better screening tests. Thyroid cancer (TC) is the most common cancer of the endocrine system and currently lacks biomarkers to detect the early stages of TC, instead, diagnosis is achieved using invasive procedures [136]. IgG N-glycan profiles from TC patients have been assayed to provide a differential diagnosis of early thyroid cancer. A comparison of IgG N-glycans from early thyroid cancer patients, healthy controls, and patients with benign thyroid nodules led to an AUC of 0.809 driven by an increase in bisecting, non-sialylated N-glycans [90].

### 6.3. Colorectal Cancer

Early colorectal cancer (CRC) arises from precancerous advanced colonic adenomas (PACA) [137]. Noninvasive, early biomarkers of colorectal cancer are urgently needed to initiate treatment for CRC patients and prevent metastasis. IgG N-glycan profiles could discriminate healthy controls from PACA (AUC = 0.84, sensitivity 61%, specificity 85%) and healthy controls from CRC (AUC = 0.84, sensitivity 72%, specificity 87%) [91]. IgG N-glycans discrimination of healthy controls compared to CRC patients was improved when combined with the traditional carcinoembryonic antigen (CEA) CRC marker. The IgG N-glycan profiling method outperformed two other noninvasive CRC screening methods, septin9 [138] and FIT [139]. Moreover, CRC patients who would experience a postoperative CRC relapse were discriminated from postoperative non-relapsing CRC patients with an AUC of 0.87 using the IgG N-glycan profiling method.

### 6.4. Pancreatic Cancer

Differentiation between pancreatic ductal adenocarcinoma (PDAC) and autoimmune pancreatitis (AIP) can be very difficult because both diseases share clinical, biochemical, and imaging characteristics [140]. Yet, IgG N-glycans discriminated PDAC from AIP with a diagnostic accuracy of 93% using a classification and regression tree (CART) validated with a second blinded cohort [85]. The group reported increased agalactosylation of IgG1 in the PDAC cohort which contrasted with increased sialylation and fucosylation ratios detected in the AIP cohort. Another study of pancreatic cancer IgG N-glycans detected early stages of the disease with an AUC of 0.91 while the traditional cancer antigen 19-9 (CA19-9) biomarker of PDAC obtained an AUC of 0.81 [93].

### 6.5. B-Cell Cancer

Lastly, IgG N-glycan profiles also change during B-cell cancers. Stages of plasma cell disorders leading to multiple myeloma begin with monoclonal gammopathy of undetermined significance (MUGS), progressing to smoldering myeloma (SMM), and lastly to multiple myeloma (MM). The IgG N-glycans present at each of these stages was found to correlate disease severity with increased agalactosylated and afucosylated N-glycan content [92]. MM in remission appeared to have recovered more of the normal galactose and fucose content, closely correlating with the traditional M-protein biomarker of MM. The agalactose content subsequently increased during MM relapse. In the SMM stage, more galactose and sialic acid was observed on IgG Fc regions compared to either MUGS or MM. Taken together, IgG N-glycan profiles can stage plasma cell disorder disease and identify patients who relapse following treatment.

## 7. Limitations

Multiple platforms have been developed to detect and quantify IgG glycosylation including MALDI MS, glycopeptide MS/MS, lectin arrays, lectin blots, UPLC-FLR, HPLC-FLR, and capillary electrophoresis. Each platform strikes a balance between throughput and sensitivity, thus comparisons of IgG N-glycan data across platforms must first consider the platform employed. In many cases, the trends observed in IgG N-glycosylation changes during a disease state will be reproduced across platforms, but the exact percentage of each N-glycan and the limit of detection will differ. In addition, there are multiple methods to isolate IgG N-glycans. Furthermore, investigators can analyze IgG from (serum plasma CSF, or mucosal sources), antigen-specific, subclass (e.g., IgG1, IgG2/3, IgG4), the Fab region, or the Fc region.

## 8. Future Perspectives

Developing clinically relevant IgG N-glycan diagnostics and prognostics will require powered clinical studies, machine learning algorithms, high-throughput methods to detect antigen-specific glycosylation, the availability of analytic platforms in clinical laboratories, and the education of clinical professionals. Parameters such as patient history, symptoms, age, weight, and sex should be combined with patient IgG N-glycosylation profiles and other biomarkers to achieve high sensitivity and specificity. The cost of IgG N-glycan diagnostics and prognostics will be determined by the amount of manual sample processing required to isolate, purify, and detect antigen-specific IgG N-glycans. A significant barrier to implementation is the need to develop an antigen-specific IgG N-glycan analytical protocol that fits into existing clinical laboratory workflows. This would allow the diagnostic to be offered at large clinical laboratories nationwide without requiring additional glycan-specific training. Diagnostics using IgG N-glycans in the United States could be offered as a laboratory-developed test (LDT), a 510K Food and Drug Administration (FDA)-cleared test, or through an FDA de novo review to classify the diagnostic as a novel or breakthrough device. FDA submissions using a 510 (k) might meet resistance due to the novelty and lack of a predicate device with similar technological characteristics. Requesting a de novo review to classify a novel device or submission as a breakthrough device if a disease is an alternative route to FDA clearance.

IgG N-glycans have the potential not only to serve as a diagnostic but also as a target to modulate autoimmune pathogenesis. Recent reports focus on treating autoimmune disease with therapeutic intravenous bulk IgG containing increased levels of sialic acid on the Fc region [141]. More research should be devoted to identifying compounds that modulate host plasmablast glycosyltransferase expression to alter immunologic responses during disease states and autoimmunity [56,142].

## 9. Conclusions

The goal of this review is to highlight the multitude of reports that apply IgG N-glycans to the diagnosis and prognosis of disease states in humans. Of the reported diagnostic performances using IgG N-glycans to distinguish healthy control cohorts from the disease state cohort, AUC ranged from 0.65 to 0.99, sensitivity ranged from 60% to 100%, and specificity ranged from 62% to 100%. Many of the diseases in this review currently lack sensitive and specific diagnostics due to overlapping symptomology with other diseases (autoimmune pancreatitis versus pancreatic ductal adenocarcinoma), a lack of tell-tale symptoms during early stages of disease (cancers and neurodegenerative disease), or limited clinical indicators of underlying disease (active versus latent tuberculosis). Thus, IgG N-glycan profiles not only provide insight into the immunological response to disease, but also have the potential to diagnose them earlier than other biomarkers.

While many of the N-glycan profiles observed during cancer, inflammation, and autoimmunity present a general increase in agalactose content with concomitant decreases in core-fucosylation, the clinical utility of using IgG N-glycans to detect disease is still feasible. Disease states reported in this literature review were discriminated from healthy controls using unique combinations of the 24 IgG N-glycan profile and the degree of the detected change. Moreover, log ratios of N-glycans and their associated classes (mono-galactosylated versus di-galactosylated, etc.) expand the number of variables to compare cohorts of disease states. As discussed, Iwamura et al. discriminated between nine urological diseases using serum immunoglobulin N-glycans processed with a disease-specific scoring system developed using a machine learning algorithm. In addition, once a specific disease state is associated with an IgG N-glycan profile, a patient’s response to treatment can be tracked longitudinally, serving as a biomarker of disease resolution.

Examining the data in this review reveals interesting associations across diseases. Lowered core-fucose content on IgG leads to increased FcγR-mediated ADCC. Accordingly, core-fucose levels in most disease states decrease except in HIV, idiopathic membranous nephropathy, and Crohn's disease. Agalactose content generally increases or does not change compared to healthy control cohorts apart from acute Lyme disease, autoimmune pancreatitis, and adult HIV patients likely to experience viral rebound after discontinuing ART therapy. Galactose and sialic acid content varied across disease states but often either increased or decreased in the same direction except in adults and children with ART-suppressed HIV. These disparate diseases sharing similar IgG N-glycan trends hint at underlying mechanisms of host immune responses and should be further interrogated by examining the expression of plasmablast glycosyltransferases and associated cytokines controlling these expression alterations. Taken together, the field of IgG N-glycomics is ripe for discovery, exploration, and innovation.

## Figures and Tables

**Table 1:** IgG N-glycan Profile Trends and Diagnostic Performance. F = core-fucose, Ag = Agalactose, G = Galactose, S = Sialic Acid, B = Bisecting, and M/H = Mannose/Hybrid N-glycans. Up and down arrows indicate if the glycan class is increased or decreased during the disease state. An arrow and a dash indicate an increase or no change while up and down arrows separated by a slash indicates specific glycans within the class increased while others decrease. Orange coloration indicates a proinflammatory effector function while green coloration indicates an anti-inflammatory effector function.

General Immune State	Condition, IgG Source	Diagnostic/Prognostic Performance	F	Aga	G	S	B	M/H	Ref.
Acute Bacterial Infection	Pediatric meningococcal sepsis	-	↓	-	-	↓	-	↑	[52]
Acute Bacterial Infection	Acute Lyme disease (LD)	LD vs healthy control (HC): Sen 75%, Spec 100%	-	↓	↑	-	-	-	[67]
Acute Viral Infection	Influenza	-	↓	-	-	↑	-	-	[68]
Acute Viral Infection	Pediatric RSV infection, ag-specific IgG	-	↓	-	-	-	-	-	[69]
Acute Viral Infection	Severe COVID-19	Severe vs mild COVID-19 prognosis: AUC 0.72	↓	↑	↓	↓	↑/↓	-	[28,68,70,71,72,73,74]
Acute Viral Infection	Dengue virus (DENV)	Fucose predicts infant DENV susceptibility	↓	↑	-	↓	-	-	[75,76,77]
Autoimmune	Vasculitis IgG1 patients likely to relapse	Vasculitis relapse vs non-relapse: AUC 0.65	-	↑	↓	↓	-	-	[22]
Autoimmune	Guillain-Barré syndrome	-	-	-	↓	↓	-	-	[78]
Autoimmune	Systemic erythematosus lupus (SLE)	SLE vs HC: Sen 80%, Spec 80%, AUC 0.84	↓	↑	↓	-	↑	-	[79]
Autoimmune	Crohn's disease (CD)	CD vs UC: Sen 60%, Spec 80%, AUC 0.75	↑	↑	-	-	-	-	[80]
Autoimmune	Ulcerative colitis (UC)	UC vs CD: Sen 60%, Spec 80%, AUC 0.75	↓	↑	-	-	-	-	[80]
Autoimmune	Immune thrombocytopenia (ITP) IgG4	IMN vs HC: Sen 80%, Spec 100%, AUC 0.96	↓	-	↓	-	-	-	[81]
Autoimmune	Chronic inflammatory airway disease	Severe asthma/sarcoidosis vs HC: AUC 0.83	-	↑	↓	-	-	-	[82]
Autoimmune	Idiopathic membranous nephropathy (IMN)	-	↑	-	↓	-	-	-	[83,84]
Autoimmune	Autoimmune pancreatitis (AIP)	AIP vs PDAC: Sen 94% Spec 93% AUC: 0.93	-	↓	-	-	-	-	[85]
Cancer	Renal cell carcinoma (RCC)	RCC vs HC, UTI, CRPC: Sen 100%, Spec 100%, AUC 0.99	-	-	-	↑	↑	-	[86]
Cancer	Castration resistant prostate cancer (CRPC)	CRPC vs HC, UTI, RCC: Sen 90%, Spec 95%, AUC 0.96	-	-	-	↑	-	-	[86]
Cancer	Stage II breast cancer	Stage II breast cancer vs HC: AUC 0.92	-	↑	-	-	-	-	[87]
Cancer	Endometrial cancer (EC)	EC vs HC: AUC 0.87	-	-	↓	↓	-	-	[88]
Cancer	Cervical intraepithelial neoplasia I (CIN I)	CIN I vs HC: Sen 73%, Spec 62%	↓	-	↑	-	-	-	[89]
Cancer	Early thyroid cancer (ETC)	ETC vs HC: AUC 0.81	-	↑	↑	-	↑	-	[90]
Cancer	Precancerous advanced colonic adenomas (PACA)	PACA vs HC: Sen 61%, Spec 85%, AUC 0.84	-	-	↑	↑	↑	-	[91]
Cancer	Colorectal cancer (CRC)	CRC vs HC: Sen 72%, Spec 87%, AUC: 0.84	-	↑	↓	↓	-	-	[91]
Cancer	Multiple myeloma (MM)	MM trends to healthy normal in remission	↓	↑	-	-	-	-	[92]
Cancer	Pancreatic ductal adenocarcinoma (PDAC)	PDAC vs HC: AUC 0.91	-	↑	-	-	-	-	[85,93]
Cancer	Cervical cancer (CC)	CC vs HC: Sens 87%, Spec 72%	↓	-	↓	-	-	-	[89]
Chronic Bact. Infection	Active tuberculosis (ATB)	ATB vs HC: Sen 76%, Spec 71%	-	↑	↓	-	-	-	[94,95,96]
Chronic Viral Infection	Adult HIV shorter viral rebound	-	-	↓	↑	-	↑	-	[97]
Chronic Viral Infection	HIV unsuppressed	-	↑	↑	-	↓	-	-	[98]
Chronic Viral Infection	HIV + ART suppression	-	↑/-	-	↑	↓	-	-	[98]
Chronic Viral Infection	Pediatric HIV + ART	-	↑/-	↑	↓	↑	↓	-	[99]
Chronic Viral Infection	Hepatitis B chronic untreated	-	-	↑	↓	↓	-	-	[100]
Inflammatory	Urinary tract infection (UTI)	UTI vs HC, RCC, CRPC: Sen 95%, Spec 95%, AUC 0.95	-	↑	↓	-	-	-	[86]
Inflammatory/Neurodegenerative	Amyotrophic lateral sclerosis (ALS)	-	↓	-	-	-	-	-	[101]
Inflammatory/Neurodegenerative	Amyotrophic lateral sclerosis (ALS)	ALS vs HC: AUC 0.79	-	-	↑	-	-	-	[102]
Inflammatory/Neurodegenerative	Parkinson's disease	Parkinson's disease vs HC: Sen 87%, Spec 92%	-	-	↑	-	-	↓	[103]
Liver Cirrhosis	Severe liver fibrosis	Severe liver fibrosis vs HC: Sen 94%, Spec 90%	-	↑	-	-	↑	-	[104]
Parasitic Infection	Naturally acquired malaria, Ag-specific IgG	-	↓	-	-	-	-	-	[105]
Pre-Cancer	Smoldering myeloma (SMM)	-	-	-	↑	↑	-	-	[92]

## Data Availability

Not applicable.
